# Recombinant expression and characterization of Canine circovirus capsid protein for diagnosis

**DOI:** 10.3389/fvets.2024.1363524

**Published:** 2024-04-10

**Authors:** Wichan Dankaona, Pornpiroon Nooroong, Napassorn Poolsawat, Chutchai Piewbang, Somporn Techangamsuwan, Panat Anuracpreeda

**Affiliations:** ^1^Department of Pathology, Faculty of Veterinary Science, Chulalongkorn University, Bangkok, Thailand; ^2^Animal Virome and Diagnostic Development Research Unit, Faculty of Veterinary Science, Chulalongkorn University, Bangkok, Thailand; ^3^Parasitology Research Laboratory (PRL), Institute of Molecular Biosciences, Mahidol University, Nakhon Pathom, Thailand

**Keywords:** antibody, canine circovirus, capsid protein, immunohistochemistry, recombinant protein, respiratory disease

## Abstract

Canine circovirus (CanineCV) is a contagious virus that causes severe gastroenteritis, diarrhea, respiratory disease, and vasculitis, often resulting in fatality among infected dogs. In this study, a recombinant Capsid protein (rCap) of CanineCV was expressed in the *Escherichia coli* (*E. coli*) Rosetta (DE3) pLysS host cell, followed by affinity purification, and then analyzed by SDS-PAGE, revealing a molecular weight of approximately 31 kDa. The antigenicity of the CanineCV rCap protein was confirmed through recognition by a rabbit anti-CanineCV rCap protein polyclonal antibody (PoAb). Additionally, the reactivity and specificity of this PoAb were assessed using indirect enzyme-linked immunosorbent assay (ELISA) and Western blot analysis before applying in an immunohistochemistry (IHC), namely, immunoperoxidase detection. The immunoperoxidase assay using rabbit anti-CanineCV rCap protein PoAb demonstrated that the CanineCV Cap protein was predominantly located in immune cells, especially lymphocytes and macrophages, within the spleen, lung, tracheobronchial lymph nodes, small intestine, and kidney. Similarly, the Cap protein was also found in pneumocytes in the lung and renal tubular epithelial cells in the kidney. These findings reflected the biological activity and cell tropism of the virus. Therefore, the recombinant Cap protein and its PoAb could be used for the development of a valuable diagnostic tool for CanineCV detection.

## 1 Introduction

Canine circovirus (CanineCV) is a non-enveloped, closed circular, single-stranded DNA (ssDNA) virus belonging to the genus *Circovirus* in the family *Circoviridae*. The CanineCV DNA has a genome length of 2,063–2,064 base pairs (bp) and contains two major open reading frames (ORFs): ORF1 and ORF2. ORF1 encodes the replication-associated (Rep) protein (303 amino acids), required for viral replication, while ORF2 encodes the major structural capsid (Cap) protein (270 amino acids). CanineCV associated with hemorrhagic gastroenteritis and diarrhea has been well reported in many countries worldwide, such as Germany (3.64–20.1%) (1, 2), Italy (32.42%) (3), the United States (11.3%) (4), China (5.6%) (5), and Taiwan (28%) (6). It can lead to fatalities in infected dogs, particularly in critical instances (1, 2, 4, 6–8). In addition to gastrointestinal tract problems, CanineCV is frequently found in oronasal secretions of dogs with respiratory diseases, such as pneumonia, and in severe cases, it can have fatal outcomes for the dogs (9–11). In several cases, CanineCV has been histologically detected in the macrophages and lymphocytes in the lymphoid tissues, such as the tonsil, spleen, lymph nodes, and Peyer’s patch in the ileum (4, 8, 10). The exact role of the virus in these cells remains uncertain. Only one published article has shown the suppression of the interferon response in the cells infected with CanineCV (12).

The *Cap* gene of CanineCV encodes the Cap protein, which is a major structural protein of the virus involving virus particle assembly. The antigenic potential of Cap protein in host immunity has been identified through an analysis of T-cell and B-cell epitopes (13, 14) and antibody levels in sera from CanineCV-infected dogs (15).

Currently, the methods used to detect CanineCV infection include conventional polymerase chain reaction (cPCR), real-time PCR (qPCR), *in situ* hybridization (ISH), transmission electron microscopy (TEM), and indirect enzyme-linked immunosorbent assay (indirect ELISA) (4, 15, 16). The ISH provides cellular localization of the virus accompanying histopathological lesions, but the procedure is slightly technically complex and costly. In comparison, the immunohistochemistry (IHC) method is less complex, inexpensive, and easy to optimize. It is also effective in identifying the virus in the presence of histopathological abnormalities. However, an IHC technique for CanineCV detection has not been established due to the unavailability of antibodies. Therefore, the study aimed to express and characterize the recombinant capsid protein (rCap protein) of CanineCV and produce a specific polyclonal antibody (PoAb) against rCap protein. Additionally, the obtained antibodies were used to identify the virus distribution in the CanineCV-infected tissues using the IHC method.

## 2 Materials and methods

### 2.1 Ethics statement

This study was approved by the Institutional Animal Care and Use Committee (IACUC) (Protocol No. 2031014) and the Institutional Biosafety Committee (IBC) (Protocol No. 1931036) of Chulalongkorn University, Thailand.

### 2.2 Cloning of CanineCV Capsid (*Cap*) gene

The PCR-positive CanineCV sample was obtained from a prior study (11). Briefly, nasal secretion was collected using Puritan swabs (Guilford, United States) from dogs with respiratory disease. The swab was placed in 1% (v/v) sterile phosphate buffer saline (PBS) and processed for genomic extraction using the Viral DNA/RNA extraction Kit (Geneaid Biotech Ltd., Taiwan) according to the manufacturer’s protocol. The extracted nucleic acid was initially tested for CanineCV *Rep* and *Cap* genes. Subsequently, the full-length genome of CanineCV was amplified using primers based on the acquired *Rep* and *Cap* gene sequences. The primers and conditions of cPCR were described in the previous study (11).

To construct the recombinant *Cap* gene, the primers were designed based on the *Cap* gene sequence deposited in the GenBank database (accession number ON863358). The truncated *Cap* gene was amplified using the primer that skipped the first 120 base pairs of the *Cap* gene, resulting in the removal of 40 amino acids from the N-terminal region. The forward primer was 5’-CAC CTT GAC AGC TGA TTG GC-3′, and the reverse primer was 5’TTA CAA CTG TCG ACC AGT TTC A − 3′. The reaction mixture consisted of 50 ng of DNA template, 0.5 μM of each primer, 200 μM of each deoxynucleotide triphosphate (dNTPs), 1x Phusion GC buffer, nuclease-free water, and 0.4 U Phusion® High-Fidelity DNA Polymerase (New England Biolabs, United Kingdom) to a final volume of 20 μL. The thermocycling condition was composed of initial denaturation at 98°C for 30 s, followed by 35 cycles of denaturation at 98°C for 10 s, annealing at 63°C for 20 s, extension at 72°C for 30 s, and a final extension at 72°C for 10 min. The amplified PCR products were stained with FluoroStain™ DNA Fluorescent Staining Dye (SMOBIO, Taiwan) and run in 1.5% agarose gel electrophoresis at 100 V, for 40 min. A 100-bp DNA Ladder M (MolBio™ HIMEDIA, India) was used as a marker. The specific band of PCR product was 693 bp in length of truncated *Cap* gene. Subsequently, the target amplicons in agarose gel were cut and purified using NucleoSpin Extract II (Macherey-Nagel, Germany) and submitted for sequencing to confirm the target sequences.

For ligation, a mixture of 4 μL of purified PCR product, 1 μL of salt solutions, and 1 μL of the pET100/D-TOPO^®^ vector was conducted according to the manufacturer’s protocol. The ligation mixture was transformed into chemically competent *E. coli* strain Top10 cells (Invitrogen) using the heat shock method. Then, 200 μL of transformed *E. coli* culture was spread on the Luria-Bertani (LB) agar plates containing 100 μg/mL of ampicillin and incubated at 37°C overnight. The positive bacterial clones were selected and grown in an LB medium containing ampicillin at 37°C overnight. The bacterial cultures were then extracted using a PureDireX Plasmid miniPREP Kit (Bio-Helix, Taiwan) according to the manufacturer’s protocol and submitted for sequencing before subcloning into the expression host cells.

### 2.3 Expression and purification of CanineCV rCap protein

The extracted pET100/D-TOPO^®^ vector containing the *Cap* gene was transformed into the Rosetta (DE3) pLysS chemically competent *E. coli* cells using the heat shock method. In total, 50 μL of transformed *E. coli* culture was grown on the LB agar plates containing 100 μg/mL of ampicillin and 25 μg/mL of chloramphenicol and incubated overnight at 37°C. The transformants were subsequently incubated at 37°C for 16 h. After that, 1% of overnight bacterial culture was added into the culture media and incubated at 37°C until the optical density (OD_600_) reached 0.5–0.8. Then, the Histidine (His)-tagged rCap protein was induced for expression by isopropyl β-D-1-thiogalactopyranoside (IPTG) (Merck, India) at a final concentration of 1 mM and further incubated for 4 h. The bacterial cell pellets were collected by centrifugation at 9,000 *g* for 10 min at 4°C and resuspended with 1:20 volume of lysis buffer (50 mM sodium phosphate buffer, 300 mM sodium chloride, 1 mM phenylmethylsulfonyl fluoride or PMSF, 20 μg/mL DNAse, 0.2 mg/mL lysozyme, and 0.2% Triton X-100, pH 8.0). The cells were mechanically lysed by sonication at 5 s each with 5 s intervals on ice and centrifuged at 13,000 *g* for 30 min at 4°C. Thereafter, the pellets were resuspended with 1:10 volume of binding buffer (50 mM sodium phosphate buffer, 300 mM sodium chloride, and 8 M urea, pH 8.0) and centrifuged at 13,000 *g* for 30 min at 4°C. The supernatant was collected and run through the HisTrap™ high-performance columns (GE Healthcare Life Science, United States) following the manufacturer’s protocol. Finally, the bound protein was eluted with the elution buffer (50 mM sodium phosphate buffer, 300 mM sodium chloride, 300 mM imidazole, and 8 M urea, pH 8.0). The protein concentration in each fraction was measured using Bradford’s assay (17) with bovine serum albumin (BSA) as a standard protein.

### 2.4 Identification of purified CanineCV rCap protein

The purified CanineCV rCap protein was run on a 12% sodium dodecyl sulfate–polyacrylamide gel electrophoresis (12% SDS-PAGE). The bands corresponding to the target protein were excised and sent for mass spectrometry to confirm the obtained protein by the NCBIprot database. Proteins with scores over 65 were considered to be statistically significant (*p* < 0.05). The verification of proteins included assessing peptide matches and the percentage of sequence coverage.

### 2.5 Production of polyclonal antibody (PoAb) against CanineCV rCap protein

Polyclonal anti-rCap protein was produced with the method described by Watthanadirek et al. (18, 19) and Junsiri et al. (20). In brief, a female New Zealand White rabbit was injected with 500 μg of purified rCap protein mixed with 500 μL of complete Freund’s adjuvant to a final volume of 1,000 μL via a subcutaneous route. Three boosters of 250 μg of purified rCap protein mixed with 500 μL incomplete Freund’s adjuvant to a final volume of 1,000 μL were injected at 2-week intervals via the same route. The whole blood was collected and transferred to clotted blood sterile tubes (BD Vacutainer^®^, United States) and then centrifuged at 3,000 *g* at 4°C for 15 min. The anti-sera against rCap protein were purified by ammonium sulfate precipitation followed by HiTrap Protein G HP columns purification (Sigma-Aldrich, United States) and stored at −20°C until further used. Pre-immunized rabbit sera collected prior to antigen injection served as negative controls in ELISA and Western blot assays.

### 2.6 Assay for assessing reactivity of rabbit anti-rCap protein PoAb

The ELISA method was assessed rigorously as described by Watthanadirek et al. (18, 19). Briefly, the 96-well plates (Nunc A/S, Denmark) were coated with 50 μL of 2.5 μg/mL of rCap protein diluted in coating buffer (15 mM Na_2_CO_3_, 35 mM NaHCO_3_, pH 9.6) at 4°C overnight. The wells were washed three times with washing buffer (0.05% Tween-20 in PBS buffer, PBST) and blocked with 100 μL/well of blocking buffer (1% bovine serum albumin (BSA) in PBST, pH 7.4) for 1 h at 37°C. After washing with the same buffer, 50 μL of serially diluted sera (5-fold dilution, from 1:5 to 1:9,765,625) from rCap protein-immunized rabbit were added triplicate into the wells and incubated at 37°C for 2 h. The plates were washed before adding 50 μL/well of horseradish peroxidase (HRP)-conjugated swine anti-rabbit IgG secondary antibody (Dako, Denmark) at 1:6,000 dilution in blocking buffer and incubated at 37°C for 1 h. After washing, the reaction was detected by incubation with 3,3′,5,5′-tetramethylbenzidine (TMB) substrate (KPL, United State) for 10 min at room temperature, followed by adding 1 N HCl to stop the reaction. The OD value at 450 nm was measured using a microplate reader (Multiskan Ascent, Labsystems, Helsinki, Finland). The OD cut-off values were calculated by the mean OD of negative samples plus 3 standard deviations (SD). An OD above this cutoff value was considered positive.

### 2.7 Western blot analysis

The Western blot was performed as described by Watthanadirek et al. (18, 19). In brief, the purified rCap proteins were run separately by 12% SDS-PAGE and transferred onto nitrocellulose (NC) membranes (GE Healthcare Life Science, United States). The membranes were blocked with blocking buffer (5% BSA in PBST, pH 7.4) for 1 h at room temperature. The NC membranes were incubated in mouse anti-6xHis antibody (Abcam, United Kingdom) at 1:3,000 dilution or purified rabbit anti-CanineCV rCap protein sera at 1:10,000 dilution at 4°C overnight. The blotted membranes were washed with PBST buffer for 5 min at room temperature. Thereafter, the membranes were incubated in HRP-conjugated goat anti-mouse IgG (Agilent Dako, United States) at 1:5,000 dilution or HRP-conjugated mouse anti-rabbit IgG (Santa Cruz Biotechnology, United States) at 1:6,000 dilution for 1 h at room temperature. After washing with the same buffer, the enzymatic reaction was detected by the addition of a substrate 3,3′-diaminobenzidine (DAB) (Sigma-Aldrich, United States) for 5 min at room temperature. Finally, the reaction was stopped by adding distilled water. The CanineCV-infected dog sera (diluted at 1:100) and rabbit pre-immunized sera or CanineCV-uninfected dog sera (diluted at 1:10,000) were used as positive and negative controls, respectively. The HRP-conjugated goat anti-dog IgG (Abcam, United Kingdom) at 1:3,000 dilution and HRP-conjugated mouse anti-rabbit IgG (Santa Cruz Biotechnology, United States) at 1:6,000 dilution were also used.

The cross-reactivity of rabbit anti-CanineCV rCap protein PoAb was assessed with the recombinant Cap proteins of porcine circovirus type 2 (PCV2) and porcine circovirus type 3 (PCV3) received courtesy of Associate Professor Dr. Manakorn Sukmak, Thailand. The proteins were run through 12% SDS-PAGE and subjected to Western blot analysis as mentioned above.

### 2.8 Immunoperoxidase assay

The PCR-positive CanineCV tissue samples of dogs obtained from the previous study (11) were initially tested negative for PCV2 and PCV3 using cPCR. These tissue samples were fixed with 10% buffered formalin and processed into paraffin blocks. The formalin-fixed paraffin-embedded (FFPE) tissue blocks were cut at 4 μm thickness and placed onto positive-charged slides. Then, the sections were deparaffinized and rehydrated with a series of xylene and alcohol concentrations followed by sequential washing with distilled water and PBS. The antigen retrieval was conducted using a citrate buffer (10 mM citrate buffer, pH 6.0) in a microwave for 10 min, followed by another wash with PBS. The endogenous peroxidase within the tissue was neutralized by immersion in 3% hydrogen peroxide for 30 min at 37°C. After a PBS wash, non-specific antigen binding was diminished by incubating with 5% skim milk in PBS at 37°C for 2 h. The sections were then incubated with rabbit anti-rCap IgG PoAb (diluted at 1:3,000) at 4°C overnight in a moist chamber. After washing, the sections were incubated with rabbit-specific Envision polymer (Dako, Germany) for 45 min at room temperature, followed by adding 3,3′-diamenobenzidine (DAB). Finally, the sections were counterstained with Mayer’s hematoxylin.

In this process, a spleen section from a PCR-positive CanineCV-infected dog (10) was used as the positive control, while a lymph node section from a PCR-negative CanineCV but PCR-positive for PCV3 from a pig was employed as the negative control. The IHC universal IgG-negative control (Enzo, ADI-950-231, United States) was used as substitution control, instead of rabbit anti-rCap IgG PoAb. The brown precipitates observed within the cellular structure were interpreted as positive results.

## 3 Results

### 3.1 Cloning and sequencing of CanineCV *rCap* gene

**Figure 1:**
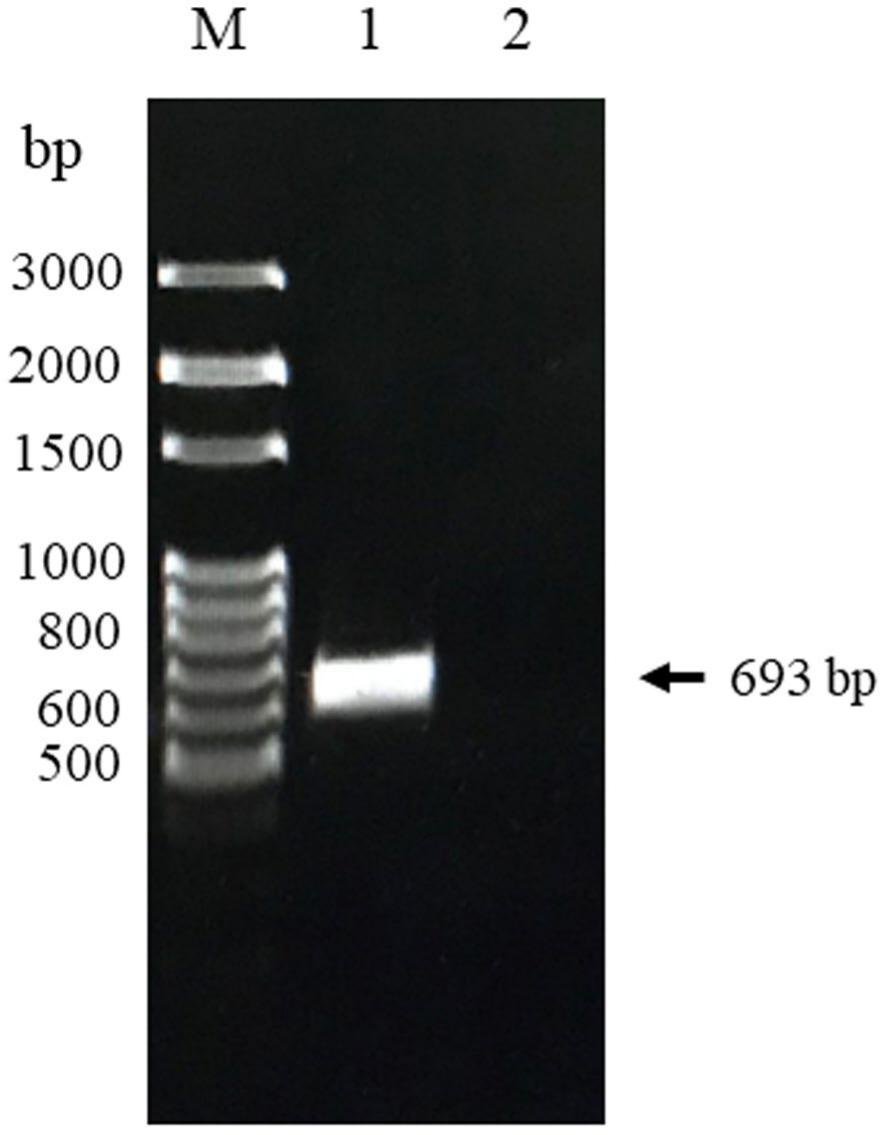
PCR product of CanineCV-truncated *Cap* gene Thailand strain obtained from nasal swab sample. Lane M: 100 bp DNA marker (MolBio™ HIMEDIA, India); Lane 1: The amplified *Cap* gene fragment; Lane 2: Negative control.

The truncated CanineCV *Cap* gene with a CACC overhang was primarily amplified by cPCR and then cloned into the pET100/D-TOPO^®^ vector to construct the recombinant plasmid. The recombinants were successfully transformed into *E. coli* Top10 cells confirmed by colony PCR and sequencing. As shown in Figure 1, the amplified PCR products from the positive samples were 693 bp in length, which was consistent with the sequencing results. There have not been any bands observed in the negative control.

### 3.2 Expression and purification of CanineCV rCap protein

**Figure 2:**
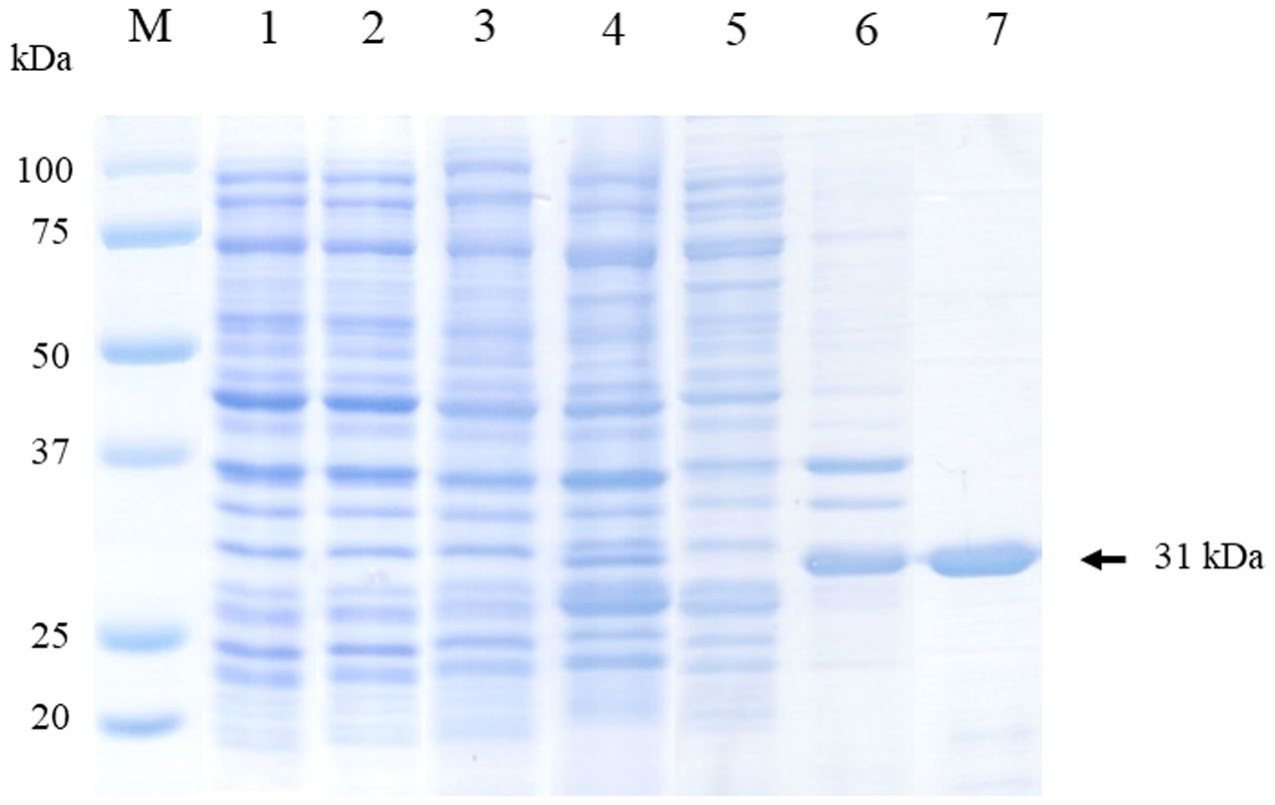
Visualization of the expression and purification of rCap protein on 12% SDS-PAGE. Lane M: Protein molecular weight marker (Bio-Rad, United States). Lane 1: Uninduced *E. coli* Rosetta (DE3) pLysS containing pET100 without *Cap*; Lane 2: Uninduced *E. coli* Rosetta (DE3) pLysS containing pET100-*Cap*; Lane 3: Induced *E. coli* Rosetta (DE3) pLysS containing pET100 without *Cap*; Lane 4: Induced *E. coli* Rosetta (DE3) pLysS containing pET100-*Cap*; Lane 5: Soluble fraction (supernatant); Lane 6: Insoluble fraction (pellet); Lane 7: Purified protein.

The CanineCV rCap protein was successfully expressed in the insoluble fraction with a molecular weight (MW) of approximately 31 kDa as shown in Figure 2. Subsequently, it was purified and exhibited a single protein band of the same size. The purified protein was confirmed through mass spectrophotometry. The result revealed that the protein band corresponded to the CanineCV rCap protein (Supplementary Table 1 and Supplementary Figure 1).

### 3.3 Reactivity and specificity of anti-CanineCV rCap protein PoAb

#### 3.3.1 Indirect ELISA

**Figure 3:**
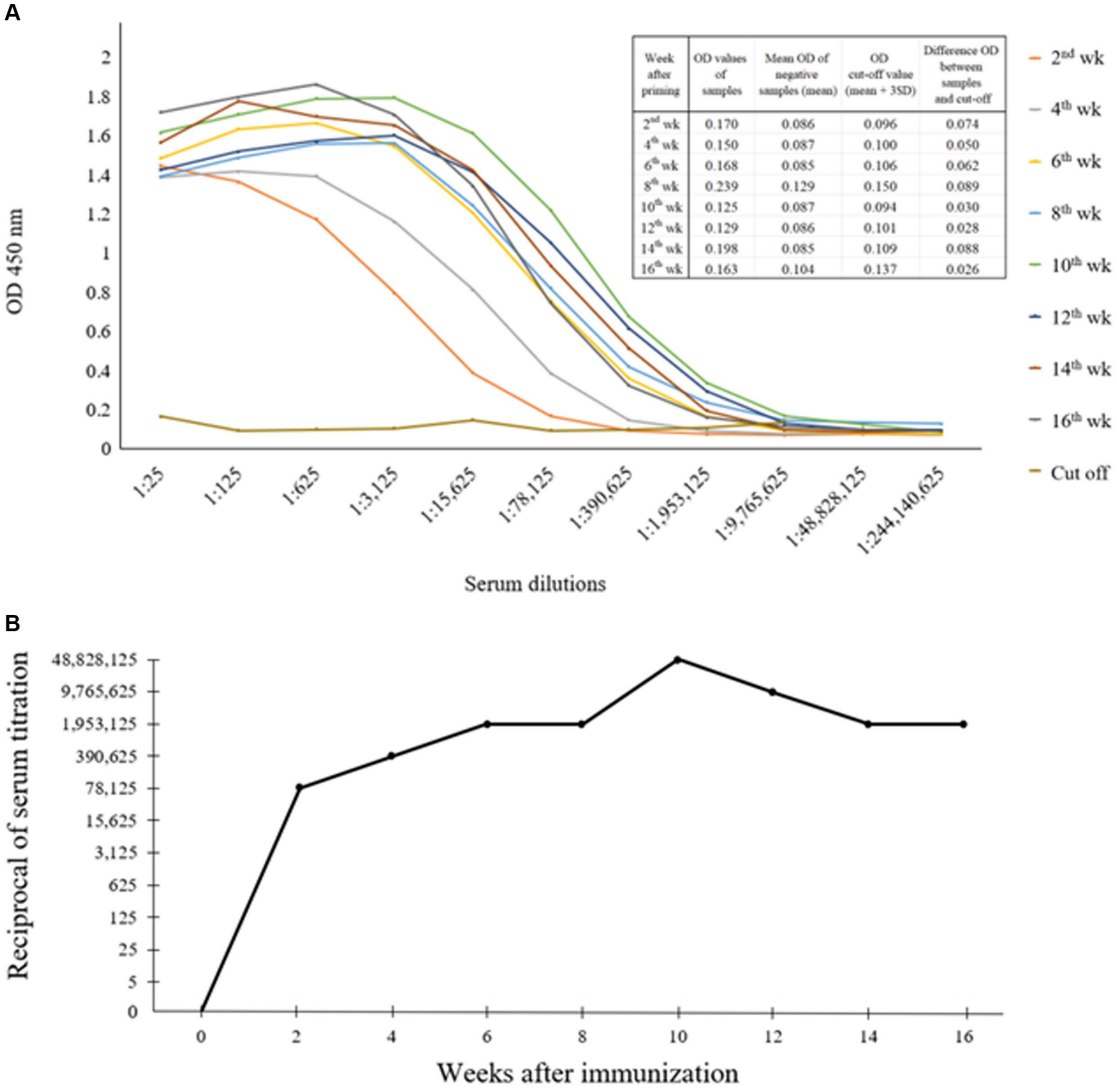
Indirect ELISA exhibited optical densities (OD) showing the levels of anti-CanineCV rCap protein antibody as observed by the various dilutions (1:25–1:244,140,625) of positive sera obtained from rabbit immunized with rCap protein **(A)**. Titer levels of anti-CanineCV antibody resulting from rabbit immunization with rCap protein **(B)**.

An indirect ELISA was conducted to evaluate the titer levels of anti-CanineCV rCap protein PoAb. As demonstrated in Figures 3A,B, the results showed that the rabbit developed an antibody response 2 weeks after priming, with a titer level of 1:78,125 and slightly increased until 8 weeks. Thereafter, the titer gradually increased to the maximum level of 1:48,828,125 at 10 weeks after priming. Then, the antibody titer decreased and maintained until the end of the blood collection period.

#### 3.3.2 Western blot analysis

**Figure 4:**
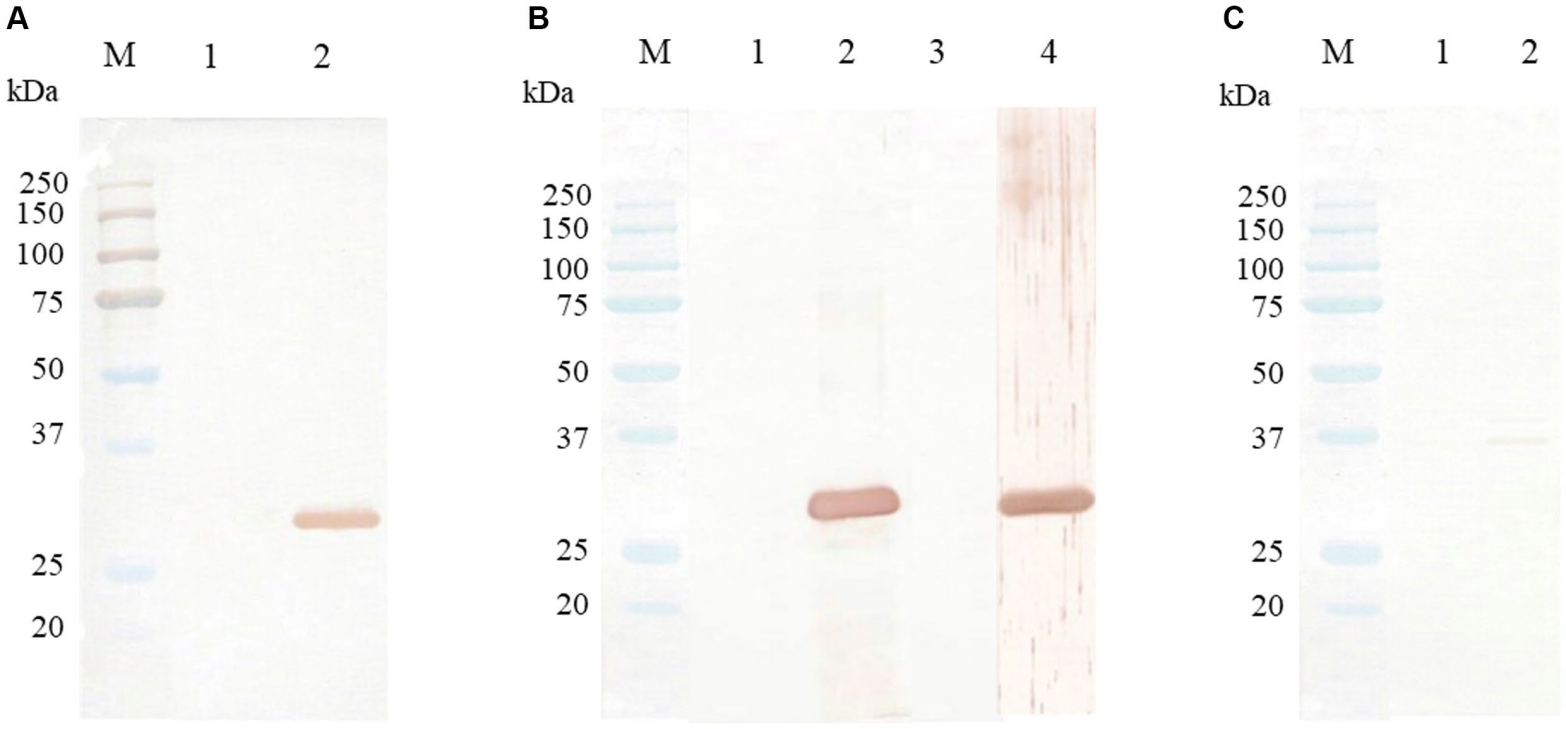
Western blot analysis of CanineCV rCap protein. Lane M is a protein molecular weight marker (Bio-Rad, United States). **(A)** Western blot patterns of CanineCV rCap protein uninduced (lane 1) and induced by IPTG (lane 2) reacted with mouse anti-6xHis tag antibody. **(B)** Western blot patterns of CanineCV rCap protein reacted with rabbit pre-immunized sera (lane 1), purified rabbit anti-rCap protein sera (lane 2), CanineCV-uninfected dog sera (lane 3), and CanineCV-infected dog sera (lane 4). **(C)** Western blot patterns of PCV2 rCap protein (lane 1) and PCV3 rCap protein (lane 2) did not react with purified rabbit anti-rCap protein sera.

The Western blot assay, using mouse anti-6xHis tag antibody, purified rabbit anti-CanineCV rCap protein sera, and CanineCV-infected dog sera, was used to evaluate the expression of CanineCV rCap protein, revealing a single band at an approximate MW of 31 kDa. Nevertheless, the negative controls, using rabbit pre-immune sera or uninfected dog sera, did not show any positive bands. Notably, rabbit anti-CanineCV rCap protein sera did not react with rCap proteins of PCV2 and PCV3 (Figure 4).

### 3.4 Distribution of CanineCV Cap protein

**Figure 5:**
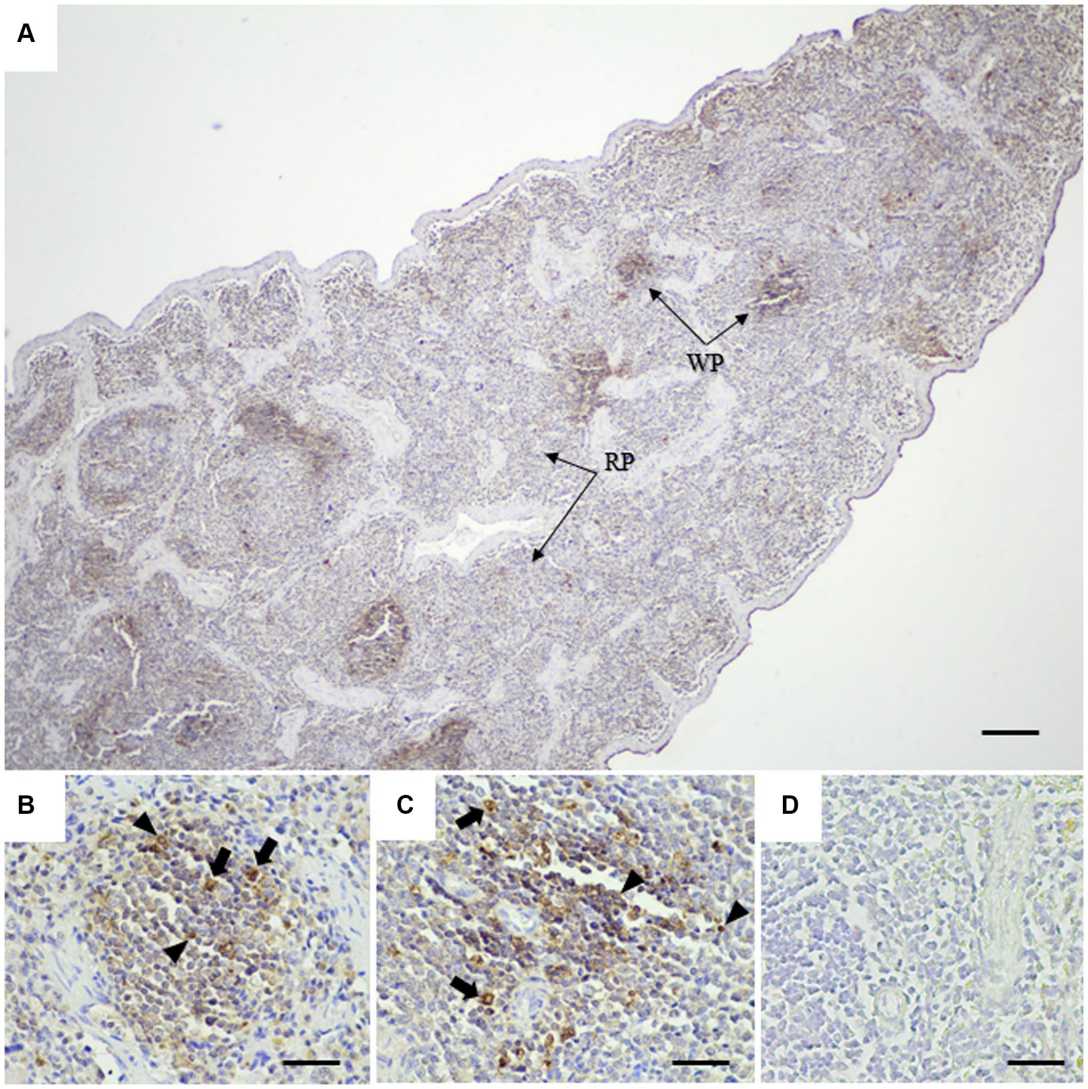
Detection of CanineCV in the spleen of PCR-positive dog by IHC. A large amount of CanineCV antigen in splenic white pulps (WP) and red pulps (RP) **(A)**. Immunolabeling of CanineCV antigen was detected in the cytoplasm of lymphocytes (arrowheads) and macrophages (arrows) within the center of lymphoid follicles **(B,C)**. No immunostaining was observed in a substitution control spleen section **(D)**. Bar = 150 μm **(A)** and 50 μm **(B–D)**.

**Figure 6:**
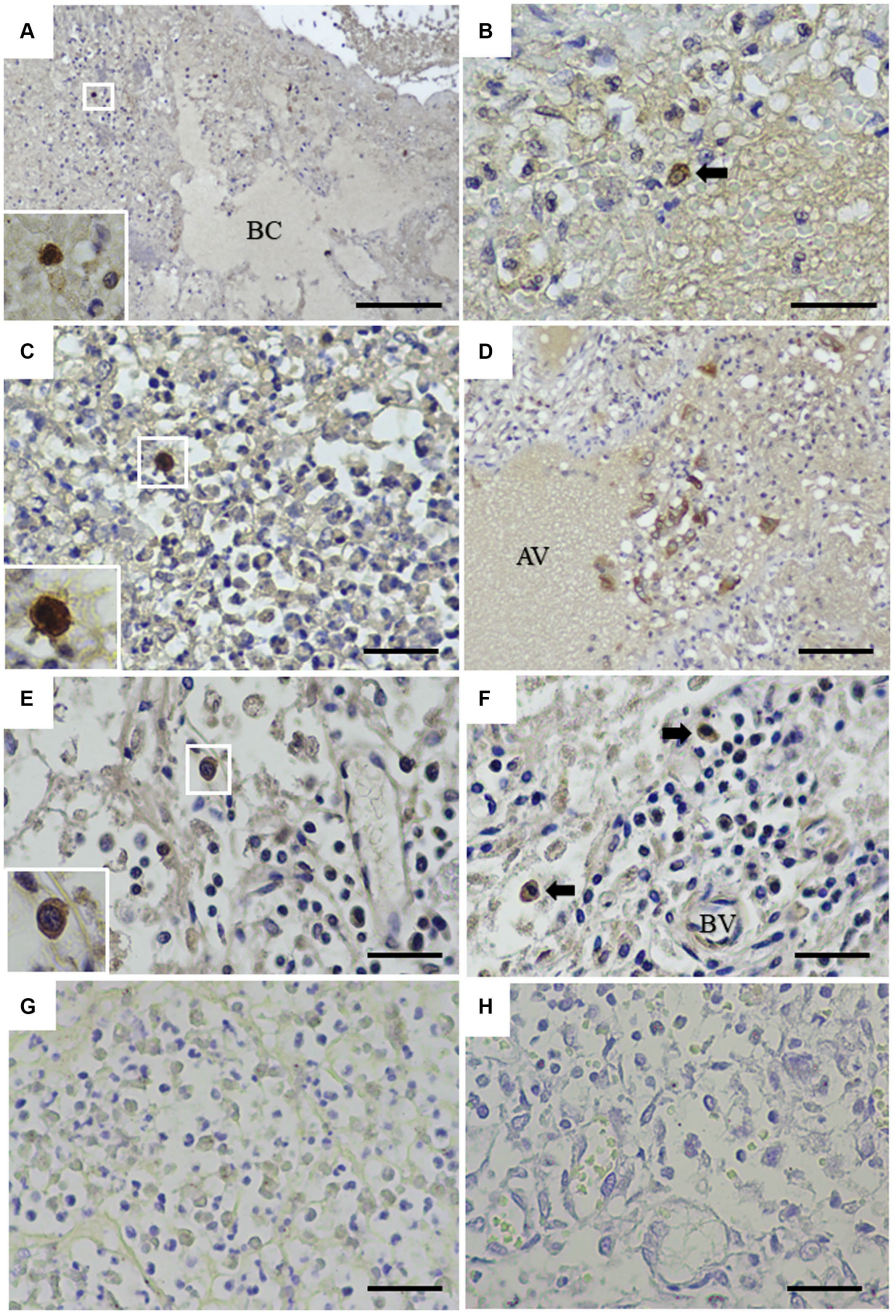
Detection of CanineCV in the lung **(A–D)** and tracheobronchial lymph nodes **(E,F)**. Intense immunostaining was detected in the cytoplasm of macrophages in the lung **(A, inset)**. Immunostaining was detected in the pulmonary alveolar macrophages-like cell (arrow) **(B)**. Macrophages in the area of suppurative inflammation contained CanineCV antigen **(C, inset)**. The pneumocytes showed positive brownish staining **(D)**. Intense stainings were demonstrated in the cytoplasm of macrophages distributed in the medullary sinuses **(E, inset)** and macrophages (arrows) surrounding blood vessels in the tracheobronchial lymph node **(F)**. No immunostaining was observed in the substitution control lung section **(G)** or the tracheobronchial lymph node section **(H)**. AV = alveolar space, BC = bronchiole, BV = blood vessel. Bar = 150 μm **(A)** and 50 μm **(B–H)**.

**Figure 7:**
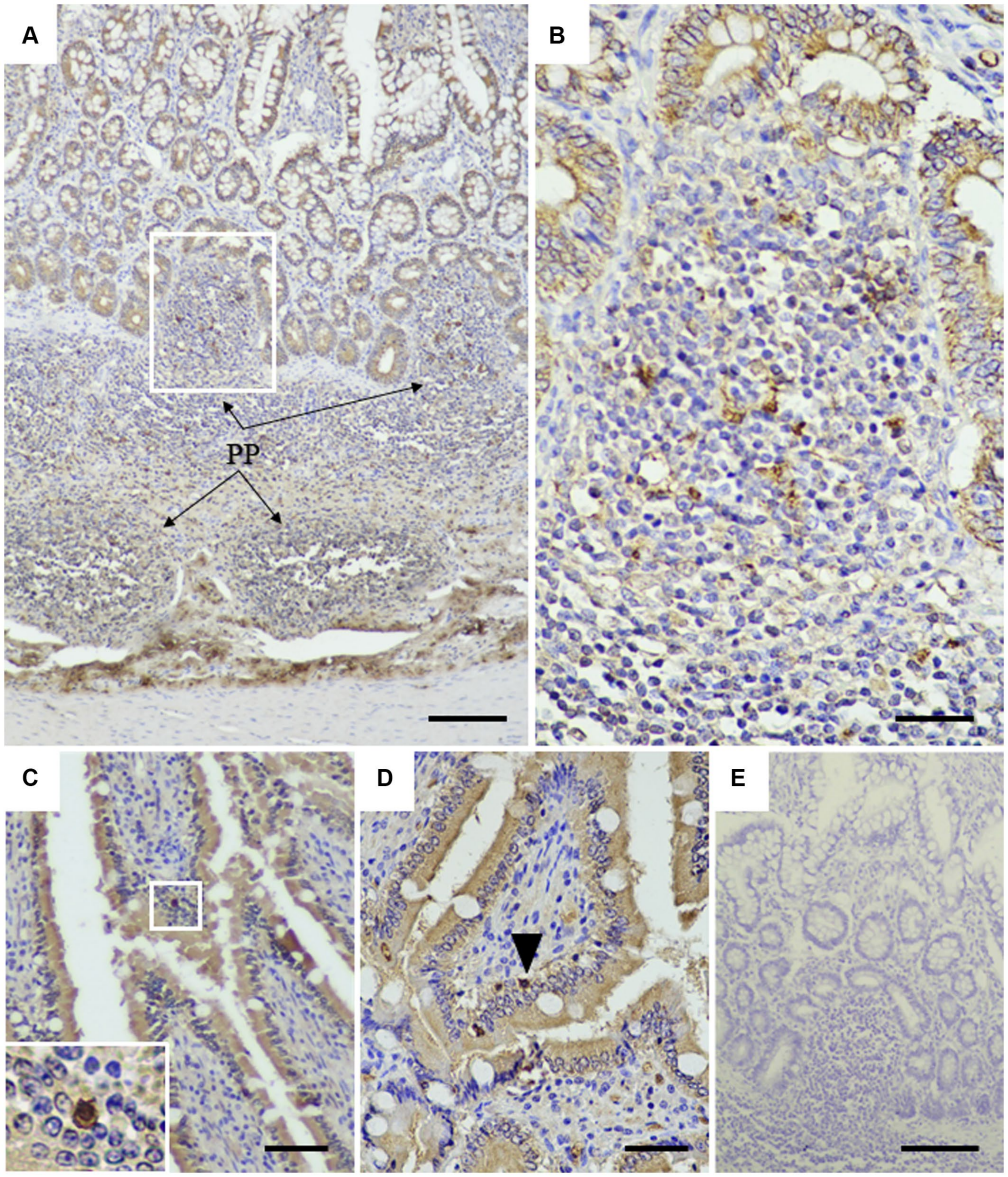
Detection of CanineCV in the ileum. Brownish immunostainings were detected in the Peyer’s patches of the ileum **(A)**. PP = Peyer’s patches. At higher magnification, in the inset of panel A, numerous mononuclear cells in the Peyer’s patches were stained positive **(B)**. Strong positive staining was observed in the nucleus and cytoplasm of plasma cells infiltrating into the intestinal villi **(C, inset)**. Positive staining of lymphocyte (arrowhead) was detected in the lamina propria layer **(D)**. No immunostaining was observed in a substitution control ileum section **(E)**. Bar = 150 μm **(A, E)** and 50 μm **(B–D)**.

**Figure 8:**
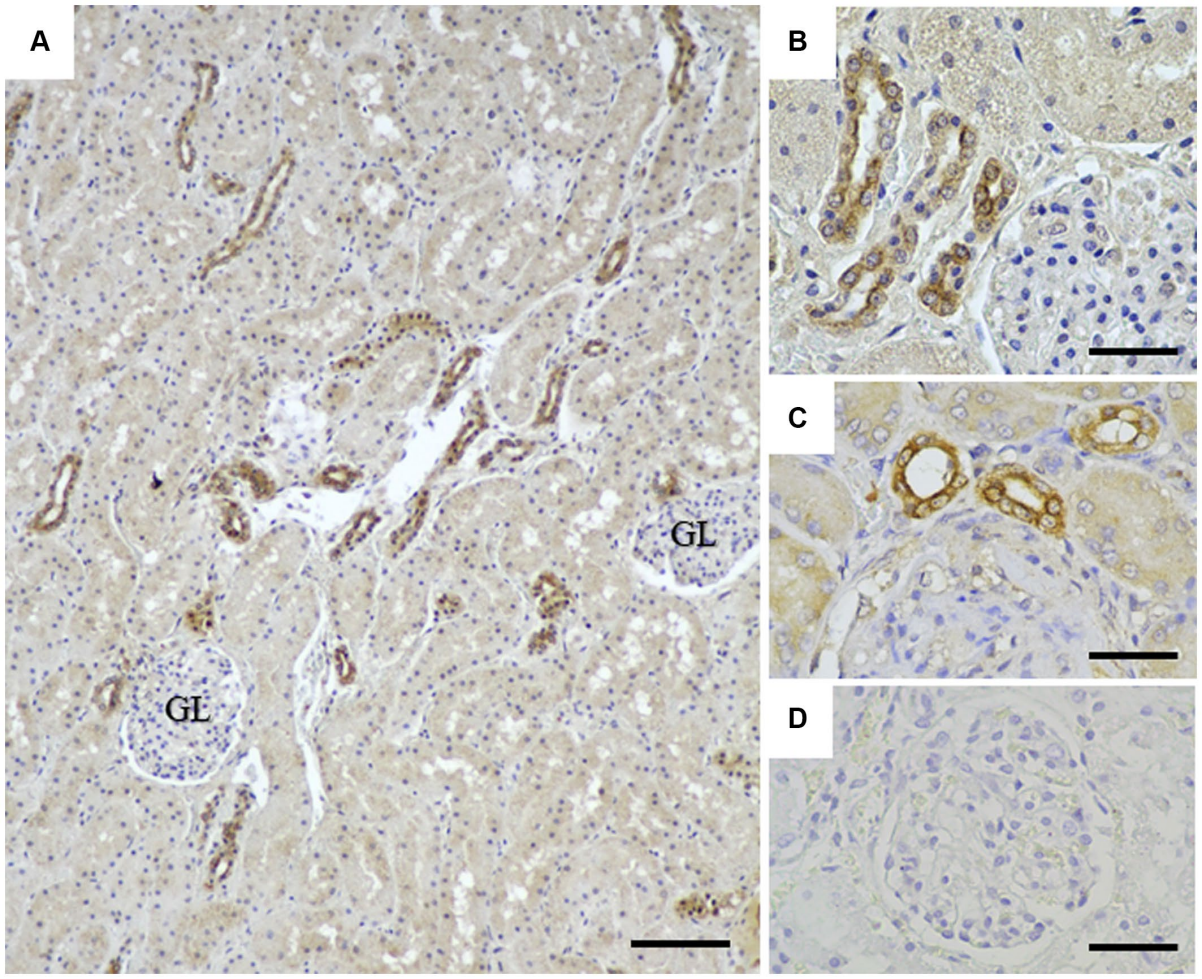
Detection of CanineCV in the kidney. Immunostainings were detected in the renal tubular epithelial cells at lower **(A)** and higher **(B,C)** magnifications. No immunostaining was observed in a substitution control kidney section **(D)**. GL = renal glomerular. Bar = 150 μm **(A)** and 50 μm **(B–D)**.

To assess the application of the rabbit anti-rCap protein PoAb, the immunoperoxidase technique was employed to detect the distribution of Cap protein in tissues from a PCR-positive CanineCV-infected dog. As presented in Figure 5, specific immunostaining was observed in the white and red pulps of the spleen (Figure 5A). In white pulps, the intense staining was mainly found in the cytoplasm of lymphocytes and macrophages (Figures 5B,C). In addition, brownish staining was observed in the cytoplasm of lymphocytes and macrophages distributed in the splenic red pulps. There was no immunostaining in the substitution control (Figure 5D) and negative control section (Supplementary Figure 2). Further identification of the CanineCV antigen in respiratory-associated organs was conducted. Positive immunostaining was shown in the lung and tracheobronchial lymph nodes (Figure 6). Intense brownish staining was detected in the cytoplasm of pulmonary alveolar macrophages residing in the lung parenchyma (Figures 6A,B), as well as in areas of suppurative inflammation (Figure 6C). Positive stainings were also apparent in the cytoplasm of type 2 pneumocytes (Figure 6D). In the tracheobronchial lymph nodes, intense immunostainings were distinctly found in the cytoplasm of macrophages (Figures 6E,F). In addition to the organ-related respiratory system, positive immunostainings were observed in the small intestine and kidney. In the small intestine section, the brownish stainings were mainly observed within the cytoplasm of mononuclear cells residing in the germinal center of the Peyer’s patch lymphoid follicle in the ileum (Figures 7A,B). A small number of positively stained macrophages and plasma cells were also found in the lamina propria layer (Figures 7C,D). Regarding the kidney section, positive stainings were detected in the renal tubular epithelial cells (Figures 8A–C). No positive signal was observed in the other tissue sections. No immunostaining was observed in the substitution control experiment in lung (Figure 6G), tracheobronchial lymph node (Figure 6H), small intestine (Figure 7E), and kidney (Figure 8D).

## 4 Discussion

In the present study, we have expressed the truncated rCap protein of CanineCV in the prokaryotic *E. coli* system. Our CanineCV rCap protein was expressed in an insoluble form, which was consistent with that of CanineCV as demonstrated by the previous report (15). This possibly may result from using the Rosetta (DE3) strain of *E. coli* as the expression host. Additionally, the low net charge of the Cap protein, which is based on the amino acid sequence may result in the expression of the Cap protein in an insoluble form (21, 22). Expressing a protein in a soluble form enables high-level production of the desired protein and toxic protein expression. This approach prevents toxic proteins that might potentially impact cell viability (23, 24). It is noteworthy in several Circovirus studies that a truncated *Cap* gene has been selected for protein expression, instead of the full-length gene (15, 25, 26). Notably, the presence of rare codons, especially arginine amino acid, is located along the Cap protein sequence and concentrates at the anterior N-terminal region of the Cap protein. As a result, the expression of the Cap protein in the *E. coli* system was decreased (27). However, these impacts can be mitigated by using *E. coli* supplied with tRNA for rare codon, Rosetta (DE3) pLysS *E. coli*, which we employed for protein expression. The successful expression of the CanineCV rCap protein was confirmed by a Western blot assay, in which the protein was identified by mouse anti-6xHis antibody, rabbit anti-serum against rCap, and the CanineCV-infected dog sera providing a protein band of 31 kDa consistent with the SDS-PAGE findings.

Because PCV2 and PCV3 can be detected in dogs (28–30) and some regions of the Cap protein share amino acid identity with PCV (<70%), the cross-reaction between our PoAb against CanineCV rCap protein and PCV might occur. By Western blot analysis, our results suggested that PoAb raised by CanineCV rCap protein-immunized rabbit specifically recognized the CanineCV rCap protein without cross-reactivity with PCV. Therefore, our first-established PoAb can be used for CanineCV detection. In this study, we did not test the cross-reactivity with proteins from other respiratory viruses that cause canine infectious respiratory disease complex (CIRDC) because the preparation of these proteins is quite difficult. However, before conducting IHC, we performed PCR testing on fresh tissues to detect the viruses as described in a previous study (11).

Currently, antibody for CanineCV detection is not yet available. In this study, we first reported the production of PoAb against CanineCV rCap protein used to detect CanineCV rCap protein in dog’s tissues using the IHC technique, such as the immunoperoxidase method. Our findings revealed that the PoAb could detect CanineCV antigen in infected tissues. Little is known regarding CanineCV-induced pathological findings in canine respiratory disease. Hence, we further investigated the presence of the virus in respiratory-associated tissues from CanineCV PCR-positive dogs that had died from respiratory disease. Our immunoperoxidase results revealed the localization and distribution of CanineCV antigen within the pneumocytes and pulmonary alveolar macrophages in areas of inflammation. In addition, immunostaining of the antigen was observed in the macrophages in the tracheobronchial lymph nodes. These pathological findings are similar to those of PCV2 (31, 32) and PCV3 (33, 34). Considering the former CanineCV study using the ISH assay, the positive signals of CanineCV genomes were detected in pneumocytes and macrophages in the lungs of dogs suffering from respiratory distress (11). The finding of CanineCV viral genomes by ISH and viral protein by IHC indicates active viral infection, providing strong support for the hypothesis of CanineCV-associated respiratory disease. Only one study demonstrated that CanineCV suppressed the antiviral response of infected cells by inhibiting type 1 interferon, which results in the enhancement of canine parvovirus replication (12). In the PCV2 study, infection of PCV2 in pulmonary alveolar macrophages disrupted the function of these cells, consequently promoting respiratory bacterial infection (35). In our cases, we speculated that the infection of these immune cells in the respiratory-associated tissues possibly interferes with and impairs the host immune response, thereby facilitating secondary bacterial infection, resulting in suppurative pneumonia (Figure 6C).

In addition to the respiratory system, a high amount of IHC signals was frequently observed in lymphocytes and macrophages in lymphoid tissues including the spleen, lymph nodes, and Peyer’s patch in the ileum, which were similar to the previous studies in CanineCV (4, 8) and PCV (34, 36, 37). The virus may have a tendency to select the lymphoid tissues, especially lymphocytes and macrophages, which may provide an advantage for virus replication as reported in PCV2 (38). However, the mechanism of the virus affecting the host immune cells needs to be explored. The present study found CanineCV antigens within the renal tubular epithelial cells. Similarly, this finding was described in porcine dermatitis and nephropathy syndrome-like disease caused by PCV3, which suggested the cell tropism for virus replication (33, 34). Nevertheless, these dogs did not have the clinical signs of renal disease, and the blood urea nitrogen and creatinine values were within normal range before death. Inoculation of the virus by *in vivo,* renal biomarkers levels monitoring, virus detection in the urine and urinary tract tissues possibly suggests the association between the virus and its effect on the canine urinary system. Additionally, this may suggest a new route for the virus to be shed, similar to the findings in a study on feline morbillivirus (FeMV), which positive immunoperoxidase results in the kidneys of cats were validated by positive PCR results in urine, urinary bladder, and kidneys in FeMV-infected cats (39).

In conclusion, we report the first production of PoAb against CanineCV rCap protein. This constructed recombinant protein exhibited apparent antigenicity and immunogenicity to stimulate immune response. The obtained PoAb, generated through immune stimulation, can specifically detect the CanineCV Cap protein, confirming the potential of the CanineCV rCap protein. Therefore, this recombinant protein can provide a foundation for vaccine production against CanineCV, and the PoAb serves as a valuable tool for immunodiagnostic purposes in further pathological studies of CanineCV infection.

## Data availability statement

The datasets presented in this study can be found in online repositories. The names of the repository/repositories and accession number(s) can be found at: https://www.ncbi.nlm.nih.gov/genbank/, ON863358.

## Ethics statement

The animal studies were approved by Chulalongkorn University Animal Care and Use Committee (No. 2031014). The studies were conducted in accordance with the local legislation and institutional requirements. Written informed consent was obtained from the owners for the participation of their animals in this study.

## Author contributions

WD: Conceptualization, Data curation, Formal analysis, Investigation, Methodology, Resources, Validation, Visualization, Writing – original draft. PN: Resources, Writing – review & editing. NP: Resources, Writing – review & editing. CP: Writing – review & editing. ST: Funding acquisition, Resources, Supervision, Writing – review & editing. PA: Data curation, Project administration, Supervision, Validation, Writing – review & editing.

## Supplementary material

SUPPLEMENTARY FIGURE 1 SDS-PAGE analysis of rCap protein. Lane M: protein molecular weight marker (Bio-Rad, USA). Lane 1: Partial purified rCap protein of PCV2; Lane 2: Partial purified rCap protein of PCV3; Lane 3: Purified rCap protein of CanineCV.

SUPPLEMENTARY FIGURE 2 Negative control for IHC. The Cap protein antigen of CanineCV was not observed in CanineCV PCR-negative lymph node sections. Bar = 150 µm (Left) and 50 µm (Right).
