# Supplementary figures and images for: Recombinant expression and characterization of Canine circovirus capsid protein for diagnosis
Source: Front Vet Sci. 2024 Apr 10;11:1363524. doi: 10.3389/fvets.2024.1363524 (PMC11040689; doi:10.3389/fvets.2024.1363524)

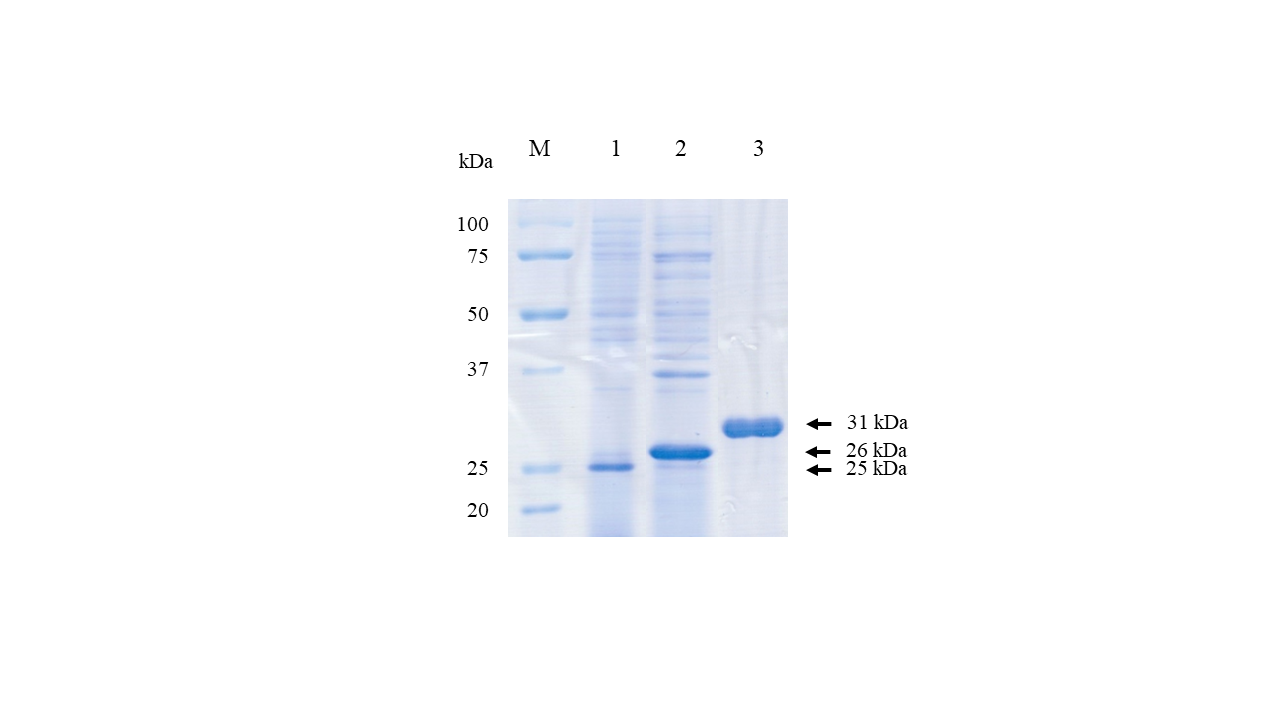

Supplement: SUPPLEMENTARY FIGURE 1 — SDS-PAGE analysis of rCap protein. Lane M: protein molecular weight marker (Bio-Rad, USA). Lane 1: Partial purified rCap protein of PCV2; Lane 2: Partial purified rCap protein of PCV3; Lane 3: Purified rCap protein of CanineCV. [file Image_1.TIF]

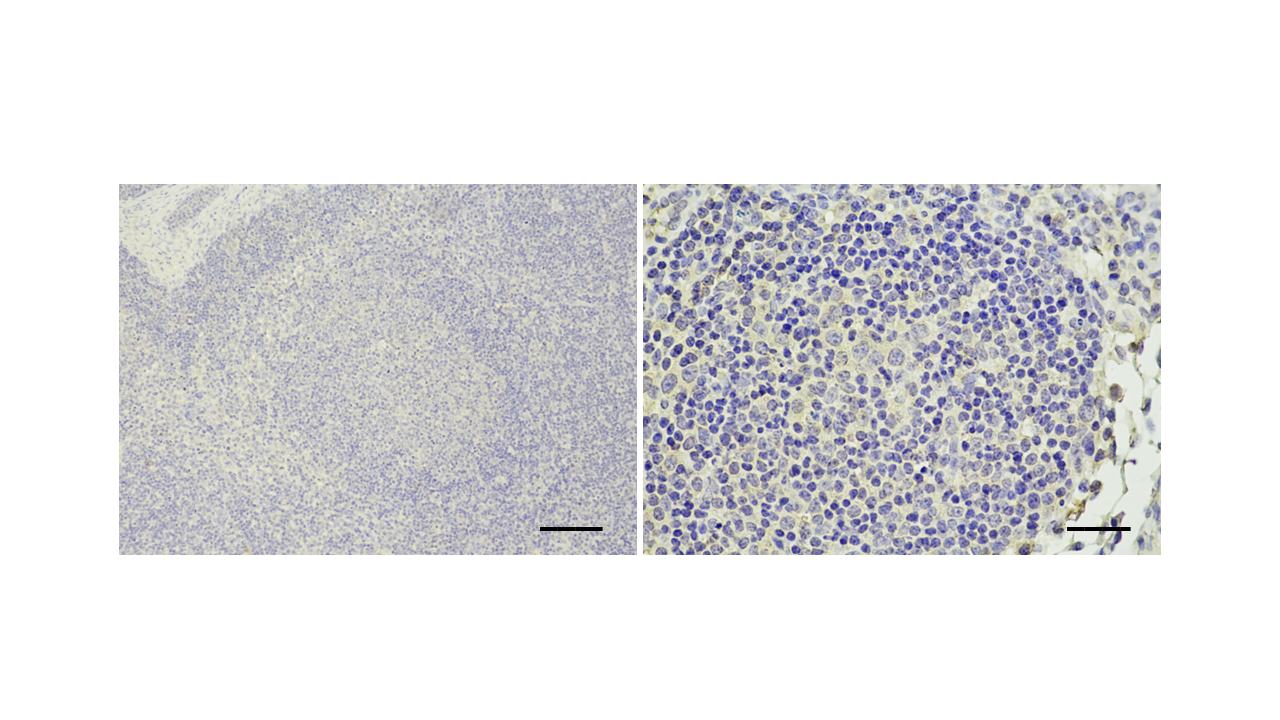

Supplement: SUPPLEMENTARY FIGURE 2 — Negative control for IHC. The Cap protein antigen of CanineCV was not observed in CanineCV PCR-negative lymph node sections. Bar = 150 µm (Left) and 50 µm (Right). [file Image_2.TIF]
