# Supplementary material for: Recombinant expression and characterization of Canine circovirus capsid protein for diagnosis
Source: Front Vet Sci. 2024 Apr 10;11:1363524. doi: 10.3389/fvets.2024.1363524 (PMC11040689; doi:10.3389/fvets.2024.1363524)
Supplement: Supplementary file 3 [file Table_1.docx]

**Supplementary Table 1** Mass spectrometry results of rCap protein

| Accession no. | Protein | Mass  (kDa) | % Coverage | No. of matched peptide sequence | m/z | Start | Stop | Matched peptide sequence |
| --- | --- | --- | --- | --- | --- | --- | --- | --- |
| A0A650AV83 | Capsid protein  CanineCV | 31.352 | 59 | 17 | 795.3855 | 73 | 93 | LTDFLQASHGTGDFQHLPPFR |
|  |  |  |  |  | 655.9691 | 106 | 120 | WINWPRTLMENVLGR |
|  |  |  |  |  | 545.2831 | 112 | 120 | TLMENVLGR |
|  |  |  |  |  | 645.2878 | 121 | 132 | TALDLDGEDQGR |
|  |  |  |  |  | 596.9495 | 121 | 137 | TALDLDGEDQGRGNATR |
|  |  |  |  |  | 544.2744 | 138 | 153 | SHLDPGTVPGIGEPPK |
|  |  |  |  |  | 685.6671 | 138 | 157 | SHLDPGTVPGIGEPPKDPNK |
|  |  |  |  |  | 591.9553 | 154 | 168 | DPNKAPFIYDPLQDR |
|  |  |  |  |  | 445.5551 | 158 | 168 | APFIYDPLQDR |
|  |  |  |  |  | 584.6158 | 158 | 172 | APFIYDPLQDRSSSR |
|  |  |  |  |  | 494.7201 | 173 | 181 | SFNMASGFK |
|  |  |  |  |  | 382.1843 | 173 | 182 | SFNMASGFKR |
|  |  |  |  |  | 697.853 | 182 | 206 | RGLTPKPMFTQDITSPSATAPWLTR |
|  |  |  |  |  | 872.7720 | 183 | 206 | GLTPKPMFTQDITSPSATAPWLTR |
|  |  |  |  |  | 1063.1747 | 233 | 260 | DMRPTTPETSTSQIPQVQYDISAYIAFK |
|  |  |  |  |  | 508.7082 | 261 | 268 | EFDYETGR |
|  |  |  |  |  | 629.2776 | 261 | 270 | EFDYETGRQL |
|  |  |  |  |  |  |  |  |  |
| Accession no. | Protein | Mass  (kDa) | % Coverage | No. of matched peptide sequence | m/z | Start | Stop | Matched peptide sequence |
| A0A5P9NYD8 | Capsid protein  CanineCV | 31.392 | 57 | 17 | 795.3855 | 73 | 93 | LTDFLQASHGTGDFQHLPPFR |
|  |  |  |  |  | 549.7393 | 112 | 120 | SLMENVLGR |
|  |  |  |  |  | 462.0245 | 112 | 132 | SLMENVLGRTALDLDGEDQGR |
|  |  |  |  |  | 645.2878 | 121 | 132 | TALDLDGEDQGR |
|  |  |  |  |  | 596.9495 | 121 | 137 | TALDLDGEDQGRGNATR |
|  |  |  |  |  | 582.6172 | 138 | 153 | SHLDPGTVPGIGEPPK |
|  |  |  |  |  | 685.6671 | 138 | 157 | SHLDPGTVPGIGEPPKDPNK |
|  |  |  |  |  | 591.9553 | 154 | 168 | DPNKAPFIYDPLQDR |
|  |  |  |  |  | 445.5551 | 158 | 168 | APFIYDPLQDR |
|  |  |  |  |  | 584.6158 | 158 | 172 | APFIYDPLQDRSSSR |
|  |  |  |  |  | 494.7201 | 173 | 181 | SFNMASGFK |
|  |  |  |  |  | 382.1843 | 173 | 182 | SFNMASGFKR |
|  |  |  |  |  | 697.853 | 182 | 206 | RGLTPKPMFTQDITSPSATAPWLTR |
|  |  |  |  |  | 872.772 | 183 | 206 | GLTPKPMFTQDITSPSATAPWLTR |
|  |  |  |  |  | 1063.1747 | 233 | 260 | DMRPTTPETSTSQIPQVQYDISAYIAFK |
|  |  |  |  |  | 508.7082 | 261 | 268 | EFDYETGR |
|  |  |  |  |  | 629.2776 | 261 | 270 | EFDYETGRQL |
|  |  |  |  |  |  |  |  |  |
|  |  |  |  |  |  |  |  |  |
| Accession no. | Protein | Mass  (kDa) | % Coverage | No. of matched peptide sequence | m/z | Start | Stop | Matched peptide sequence |
| A0A650AV31 | Capsid protein  CanineCV | 31.420 | 57 | 17 | 795.3855 | 73 | 93 | LTDFLQASHGTGDFQHLPPFR |
|  |  |  |  |  | 549.7393 | 112 | 120 | SLMENVLGR |
|  |  |  |  |  | 462.0245 | 112 | 132 | SLMENVLGRTALDLDGEDQGR |
|  |  |  |  |  | 645.2878 | 121 | 132 | TALDLDGEDQGR |
|  |  |  |  |  | 596.9495 | 121 | 137 | TALDLDGEDQGRGNATR |
|  |  |  |  |  | 582.6172 | 138 | 153 | SHLDPGTVPGIGEPPK |
|  |  |  |  |  | 685.6671 | 138 | 157 | SHLDPGTVPGIGEPPKDPNK |
|  |  |  |  |  | 591.9553 | 154 | 168 | DPNKAPFIYDPLQDR |
|  |  |  |  |  | 445.5551 | 158 | 168 | APFIYDPLQDR |
|  |  |  |  |  | 584.6158 | 158 | 172 | APFIYDPLQDRSSSR |
|  |  |  |  |  | 494.7201 | 173 | 181 | SFNMASGFK |
|  |  |  |  |  | 382.1843 | 173 | 182 | SFNMASGFKR |
|  |  |  |  |  | 697.853 | 182 | 206 | RGLTPKPMFTQDITSPSATAPWLTR |
|  |  |  |  |  | 872.772 | 183 | 206 | GLTPKPMFTQDITSPSATAPWLTR |
|  |  |  |  |  | 1063.1747 | 233 | 260 | DMRPTTPETSTSQIPQVQYDISAYIAFK |
|  |  |  |  |  | 508.7082 | 261 | 268 | EFDYETGR |
|  |  |  |  |  | 629.2776 | 261 | 270 | EFDYETGRQL |

**Supplementary Table 2** ELISA results showing OD_450_ values of samples, negative controls, cut-off and the difference in OD_450_ values between positive samples and cut-off values.

| Week after immunization | Dilution of serum samples | | | | | | | | | | Mean negative | SD | OD_450_ cut-off | Difference OD_450_ |
| --- | --- | --- | --- | --- | --- | --- | --- | --- | --- | --- | --- | --- | --- | --- |
|  | 1:25 | 1:125 | 1:1625 | 1:3125 | 1:15625 | 1:78125 | 1:390625 | 1:1953125 | 1:9765625 | 1:48828125 |  |  |  |  |
| 2 | 1.448 | 1.364 | 1.175 | 0.799 | 0.389 | **0.170** | 0.095 | 0.076 | 0.072 | 0.076 | 0.086 | 0.004 | 0.096 | 0.074 |
| 4 | 1.389 | 1.419 | 1.393 | 1.162 | 0.814 | 0.386 | **0.150** | 0.093 | 0.078 | 0.083 | 0.087 | 0.005 | 0.100 | 0.050 |
| 6 | 1.484 | 1.634 | 1.663 | 1.548 | 1.207 | 0.759 | 0.364 | **0.168** | 0.096 | 0.086 | 0.085 | 0.007 | 0.106 | 0.062 |
| 8 | 1.391 | 1.488 | 1.561 | 1.564 | 1.245 | 0.823 | 0.420 | **0.239** | 0.149 | 0.136 | 0.129 | 0.007 | 0.150 | 0.089 |
| 10 | 1.616 | 1.710 | 1.789 | 1.795 | 1.615 | 1.221 | 0.679 | 0.339 | 0.170 | **0.125** | 0.087 | 0.002 | 0.094 | 0.030 |
| 12 | 1.427 | 1.520 | 1.574 | 1.602 | 1.416 | 1.055 | 0.616 | 0.295 | **0.129** | 0.102 | 0.086 | 0.005 | 0.101 | 0.028 |
| 14 | 1.566 | 1.777 | 1.699 | 1.654 | 1.423 | 0.937 | 0.516 | **0.198** | 0.098 | 0.090 | 0.085 | 0.008 | 0.109 | 0.088 |
| 16 | 1.721 | 1.798 | 1.862 | 1.707 | 1.343 | 0.748 | 0.326 | **0.163** | 0.116 | 0.100 | 0.104 | 0.011 | 0.137 | 0.026 |

The first positive OD_450_ values above the cut-off values are shown in boldface.

SD = Standard deviation of negative samples, OD_450_ cut-off = mean negative + 3SD, Difference OD_450_ = OD_450_ of positive samples – OD_450_ cut-off


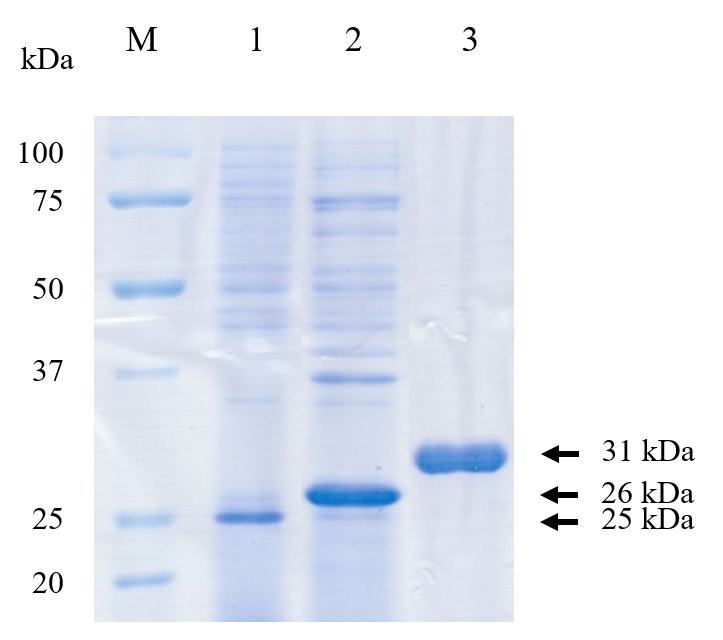


**Supplementary Figure 1.** SDS-PAGE analysis of rCap protein. Lane M: protein molecular weight marker (Bio-Rad, USA). Lane 1: Partial purified rCap protein of PCV2; Lane 2: Partial purified rCap protein of PCV3; Lane 3: Purified rCap protein of CanineCV.


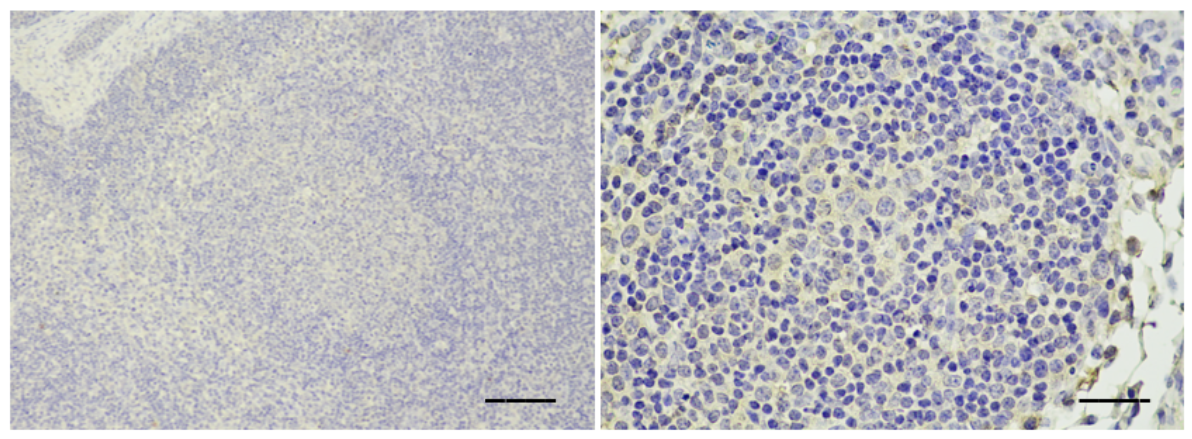


**Supplementary Figure 2.** Negative control for IHC. The Cap protein antigen of CanineCV was not observed in CanineCV PCR-negative lymph node sections. Bar = 150 µm (Left) and 50 µm (Right).
